# Newborn Feeding Knowledge and Attitudes among Medical Students

**DOI:** 10.3390/ejihpe13030043

**Published:** 2023-03-05

**Authors:** Henrique Pereira, Ricardo Campos, Patricia Silva, Madalena Cruz

**Affiliations:** 1 Department of Psychology and Education, Faculty of Social and Human Sciences, University of Beira Interior, Pólo IV, 6200-209 Covilhã, Portugal; 2 Research Centre in Sports Sciences, Health Sciences and Human Development (CIDESD), 5001-801 Vila Real, Portugal; 3 UCSP Fundão (Personalized Health Care Unit), Rua Parque Desportivo—Centro de Saúde do Fundão, 6230-411 Fundão, Portugal

**Keywords:** newborn feeding knowledge, newborn feeding attitudes, medical students

## Abstract

This study sought to assess newborn feeding knowledge and attitudes among medical students. A sample of 649 Portuguese medical students completed an online survey containing a sociodemographic questionnaire, the Newborn Feeding Ability Questionnaire (NFA), and the Iowa Infant Feeding Attitudes Scale (IIFAS). The overall sample showed moderate scores for all variables. Gender analysis identified significant differences only for the dimension related to the benefits of skin-to-skin contact between mother and newborn where women scored higher. Analysis by year of training found that students with more years of training scored higher on all variables of newborn feeding knowledge that were positively correlated and were positive predictors of newborn feeding attitudes. Students with fewer years of training scored higher on work practices interfering with newborn feeding ability, which were negatively correlated and were negative predictors of newborn feeding attitudes. These results demonstrate that medical students with more years of training are the most prepared, however, the moderate results of the sample raise concerns. Our results point to the importance of providing medical students with adequate knowledge in order to influence their attitudes toward newborn feeding and contribute to better working practices for future health professionals.

## 1. Introduction

The relationship between beliefs, knowledge, and behavior of individuals in the face of a health-related situation has been increasingly studied [1,2,3,4]. In this sense, the health belief model (HBM) points out that individuals develop their own ideas and theories about health and disease processes from the intersection of three main components: Personal factors, such as motivation, confidence, hope, fear, and ability to follow recommendations, previous experiences that strengthen beliefs on how to act when facing situations, and exposure to sources of social and cultural nature, such as learning, social influences, the media, among others. As a result, individuals develop representations that shape their habits and behaviors in relation to certain life situations, which can be beneficial or negatively influence attitudes towards them [5,6].

For the practice of newborn nutrition, this theory can also be applied [7] since knowledge and beliefs about newborn feeding may influence the parents’ attitudes and behaviors about the initial feeding process [8,9,10]. For example, mothers’ knowledge and attitudes towards breastfeeding are essential factors affecting infant feeding methods [11]. However, studies show that choosing the type of food, its duration, and continuity are influenced by several life factors, such as: The mother’s age, education level, residence (urban/rural), sociodemographic and cultural characteristics, past experiences, income level, and attitudes of the grandmother or the spouse, in addition to the expected gender roles regarding pregnancy, breastfeeding, and maternal care. Specifically in the practice of newborn nutrition, studies have been centered on the mother, highlighting the importance of the gender roles expected from women [7,12,13] in their decision-making. As such, a gender perspective analysis can reveal significant differences for the variables under study. In addition, health professionals play a vital role in promoting adequate newborn feeding practices with caregivers [14,15], and, just as individuals’ beliefs affect their health behaviors, medical practices and attitudes themselves are also influenced by the knowledge and beliefs of health professionals [16,17,18,19]. For example, Cricco-Lizza [20] investigated attitudes, beliefs, and personal experiences about breastfeeding in nursing students at the beginning of their formal work, having found that students’ positive attitudes towards breastfeeding were decisive in promoting guidance for families. Hence, having knowledge, positive attitudes, and adequate practices among health professionals are among the factors that significantly affect the success of newborn feeding.

Since health professionals are responsible for planning and implementing adequate newborn feeding practices, it is necessary that they have academic knowledge about them, which makes it possible to overcome personal beliefs so that they can implement scientifically based guidelines that are beneficial to families [21,22,23]. In this sense, it is essential that best feeding practices are taught at all levels of medical education as part of the academic curriculum [16,22,23]. This is particularly relevant as neonatal health and survival are improved by delivering essential newborn care, such as hygiene, thermal protection, initiation of breathing, and newborn feeding [24]. If this care is not provided properly, it can interfere with the health and well-being of the newborn.

However, health professionals, including medical students, do not always obtain adequate training on newborn feeding during their academic careers [16,19,25,26]. In the study by Al-Nassaj and collaborators [27], though health professionals had a positive attitude towards breastfeeding, their knowledge was insufficient or inadequate. In the systematic review by Yang et al. [28], it was evident that breastfeeding was only tackled during the maternal-infant health disciplines, and it was found that nursing, medical, and clinical students lacked knowledge about breastfeeding even after finalizing the maternal and child health study units. These results are even worse when it comes to health professionals who are in their first years of work, as well as students in the early years of study, who had less knowledge about breastfeeding management practices than those in the final years of graduation [28] and this should be taken into consideration when studying Newborn Feeding Knowledge and Attitudes among Medical Students.

Therefore, assessing the levels of knowledge and attitudes of health professionals is a decisive factor for the success of newborn feeding practices and considering that medical students, as future doctors, will be at the forefront in dealing with these questions, it is of utmost importance to understand the current situation among medical students in the Portuguese context where no Portuguese studies focusing on this topic have been found. Thus, the objective of this study is to understand newborn feeding knowledge and attitudes among Portuguese medical students. We also intend to analyze how variables, such as years of training and gender, can influence these results. This is because, as seen, knowledge of medical students can influence attitudes in their future clinical practice. We hypothesize that students with more years of training have greater knowledge and more positive attitudes towards newborn feeding, and that women participants will present more knowledge and more positive attitudes. In addition, this study could serve as a resource to identify gaps in medical training, which is an important step towards improving the work practices of future health professionals.

## 2. Materials and Methods

### 2.1. Ethical Approval

This study was approved by the Ethics Committee of the University of Beira Interior—Covilhã (Portugal) (CE-UBI-Pj-2020-083), and the Ethics Committee of the NOVA Medical School in Lisbon (Portugal) (07/2021/CEFCM).

### 2.2. Sample Selection

Inclusion criteria were the following: Being 18 years of age or older and being enrolled in a medical program from a Portuguese medical school/university. Students with intermittent enrollment and who were not able to understand Portuguese were excluded. A total of 373 from approximately 12,575 students registered in a medical program in 2020 in Portugal (data from the Contemporary Portugal Database [29]) participated in our study.

It was a convenience sample gathered online through mailing lists among medical schools and from student associations of the same schools. The surveys were made available through a webpage specifically created for the purpose of this research, and dissemination took place from December 2020 to February 2021. All ethical criteria of traditional research, such as informed consent, confidentiality, and anonymity of personal information, were considered.

### 2.3. Instrument Measures

#### 2.3.1. Sociodemographic Questionnaire

As evidenced by the health belief model (HBM) [30], individuals develop their own ideas and theories about health and disease processes from the intersection of personal factors, previous experiences, and exposure to sources of social and cultural nature. Therefore, to understand the profile of our respondents, a sociodemographic questionnaire was used comprised of the following questions: Gender, marital status, place of residence, socioeconomic status, medical school attended, year of training, attendance of hospital internship, self-perceived anxiety symptoms, self-identified problems in life, physical illness diagnosed, and mental illness diagnosed.

#### 2.3.2. The Newborn Feeding Ability Questionnaire

The Newborn Feeding Ability questionnaire (NFA) [31] was comprised of twenty-one items with responses on a 5-point Likert scale of 1 = “strongly disagree” to 5 = “strongly agree”. Items measure knowledge about (1) physiological and emotional benefits of skin-to-skin contact for newborns and mothers, (2) indicators of effective suckling and milk transfer, and (3) work practices that interfere with newborn feeding ability. The NFA has a possible total score of 105, with higher scores reflecting greater knowledge, four items are reverse scored to minimize response bias, and it is validated for the Portuguese health professional population [32]. In this study, a Cronbach’s alpha of 0.801 was obtained, indicating very good reliability.

#### 2.3.3. The Iowa Infant Feeding Attitude Scale

The Iowa infant feeding attitudes scale (IIFAS) [33] was intended to measure attitudes towards infant feeding methods and to estimate breastfeeding intention and exclusivity. The questionnaire was comprised of seventeen items with a 5-point Likert scale ranging from 1 (strongly disagree) to 5 (strongly agree). The total IIFAS score can range from 17 to 85, with higher scores reflecting positive attitudes towards breastfeeding. Total IIFAS scores can be further categorized into groups: (1) positive to breastfeeding (IIFAS score 70–85), (2) neutral (IIFAS score 49–69), and (3) positive to formula feeding (IIFAS score 17–48). In the present study, a Cronbach’s alpha of 0.771 was obtained indicating good reliability.

### 2.4. Data Analyses

For statistical analyses, we used the Statistical Package for Social Sciences (SPSS© version 27). We calculated Cronbach’s alpha by taking the score from each scale item and correlating them with the total score for each observation, and then comparing that with the variance for all individual item scores. Sociodemographic characteristics were analyzed by using absolute and relative frequencies, percentages, mean, and standard deviation. Student’s t-tests were used to compare means between two groups (genders), and ANOVAs were used to compare means of more than 6 groups (year of training). To assess the levels of association between the variables under study, Pearson correlation coefficients were implemented. To determine the predictive effects, we conducted a hierarchical linear regression. Finally, we computed the means of the 21 items of the NFA into a general measure of knowledge, which we called Overall NFA, and the means of the 17 items of the IIFAS into a general measure of attitudes, which we called Overall IIFAS.

## 3. Results

Sociodemographic characteristics of participants (*n* = 649; M_age_ = 22.45; SD = 4.08) have been described elsewhere [34]. Most were female (78.4%), single 94.5%, and lived in urban areas (78%). The respondents attended the University of Lisbon (41.4%), the University of Beira Interior (32.2%), the University of Minho (11.1%), the Institute of Biomedical Sciences—University of Porto (5.4%), the NOVA Medical School (5.2%), the University of Algarve (3.4%) and others (1.2%). 17.6% of respondents were in the 1st or 2nd year of training, around 15% were in the 3rd or 4th year, 16.2% were in the fifth year, and 19.6% were in the sixth. 64.3% said that they had already attended an internship at a hospital.

Table 1 shows results for overall scores of Newborn Feeding Knowledge, respective three sub-scales, and Attitudes. Our results show moderate scores for all assessed variables when compared to the cut-off point of 3 (expected median). This median was estimated by adding together the lower (1) and higher (5) values and dividing them by two (=3).

Table 2 displays the results for all variables by gender. Significant differences (*p* < 0.05) were obtained for “physiological and emotional benefits of skin-to-skin contact for newborns and mothers” dimension, indicating that women scored higher knowledge in this variable. Regarding other variables, the results of the gender analysis indicated moderate levels for both groups. In this sense, our respondents’ perceptions of these variables were not influenced by gender.

Table 3 demonstrates the results for all variables by year of training. In the Newborn Feeding knowledge scale, significant differences (*p* < 0.05) were found for “indicators of effective suckling and milk transfer”, where 5th and 6th year students scored higher, as well as in “work practices that interfere with newborn feeding ability”, where 1st and 2nd year students scored higher. Regarding the Overall Newborn Feeding Attitudes, last years of training scored higher.

A correlation matrix was conducted to assess association levels between the variables under study. Positive and significant associations were found (*p* < 0.05) for most variables, including “physiological and emotional benefits of skin-to-skin contact for newborns and mothers” and “year of training”. Negative and significant associations were found (*p* < 0.001) for “work practices that interfere with newborn feeding ability” dimension and “age”, “year of training” and the “Overall Newborn Feeding Attitudes”. “Overall Newborn Feeding Knowledge” and “Overall Newborn Feeding Attitudes” were significantly correlated (Table 4).

Finally, a hierarchical linear regression analysis was conducted to determine the predive effect of independent variables on Overall Newborn Feeding Attitudes. In the first block (Model I), possible confounding variables “age”, “gender”, and “year of training” were added, explaining 11.6% of overall variance. In the second block (Model II), Physiological and emotional benefits of skin-to-skin contact for newborns and mothers, Indicators of effective suckling and milk transfer, and Work practices that interfere with newborn feeding ability were added, explaining 23.4% of overall variance. Variables “Year of training”, “physiological and emotional benefits of skin-to-skin contact”, and “indicators of effective suckling and milk transfer” were positive and significant predictors of Overall Newborn Feeding Attitudes. The Work practices that interfere with newborn feeding ability were a significant negative predictor of Overall Newborn Feeding Attitudes among medical students (Table 5).

## 4. Discussion

Our study offers preliminary contributions to the understanding of newborn feeding knowledge and attitudes among medical students in Portugal. The results indicate moderate levels for all dimensions of the NFA questionnaire and for the Overall Newborn Feeding Attitudes measure among the study sample. Gender analysis revealed significant differences only for the physiological and emotional benefits of skin-to-skin contact for newborns and mothers, where women scored higher. Moreover, we found significant differences for most dimensions of the instruments when compared by year of training. Here, those with more years of training scored higher, except for work practices that interfere with newborn feeding ability. Furthermore, when evaluating the predictive effect of the Overall Newborn Feeding Attitudes, gender, age, and year of training were significant predictors, explaining 11.6% of the overall variance. However, the dimensions of the NFA questionnaire were the strongest predictors, explaining 23.4% of the overall variance. These dimensions include the students’ level of knowledge regarding different fundamental variables in the newborn feeding process, as such, the results reinforce the importance of education and training for Portuguese medical students and the impacts that their knowledge and attitudes may have on their future clinical practice of newborn feeding.

Women reported greater knowledge about the physiological and emotional benefits of skin-to-skin contact for newborns and mothers. This dimension is related to knowledge about the initial contact between the mother and the baby, as exemplified by item 15 “Separation of a newborn from the mother at birth can cause harmful stress to the baby” and item 05 “Skin-to-skin contact is important to help stabilize newborn breathing”. According to the theory of gender roles, most women are taught to be mothers from an early age, which implies the understanding that a newborn is dependent on them and that they are the only ones who can provide the care they need [35,36,37]. When these needs are not met by the mother, they may feel incapacitated, which generates feelings of guilt and shame for the perceived transgression of motherhood [38,39]. In this case, the system of internal beliefs about the mother-child relationship that the female participants have may have influenced their responses in this dimension.

In contrast, our research found no significant differences between men and women for the other study variables. This result could be linked to the fact that these variables are mainly related to the feeding process of newborns, for example, in item 02 “Newborns will develop predictable, coordinated feeding behaviors within minutes of birth” and item 14 “Midwives and mothers know the baby is getting colostrum at first breastfeed when they see the baby swallow”. In this case, in addition to the belief system, students are influenced by their own academic learning, which could have equally and moderately influenced the answers of both men and women in this sample. In fact, the academic curriculum of medical students in Portugal is structured globally and applied equally by students, regardless of gender [40]. Thus, our participants, men and women alike, enrolled in a Portuguese medical educational institution, seem to have access to the same academic curriculum and level of knowledge.

When we compared the results between the medical students’ years of training, statistically significant differences for the study variables were found. Students with more years of study (5th and 6th years) scored higher in three of the four dimensions evaluated in the NFA questionnaire, showing significant differences in terms of indicators of effective suckling and milk transfer and in the Overall Newborn Feeding Attitudes. These results were expected due to the fact that these dimensions are more related to knowledge about the feeding process itself and that, in turn, may influence the professionals’ attitudes. Furthermore, we found a positive correlation between levels of Overall Newborn Feeding Knowledge and Overall Newborn Feeding Attitudes and found that knowledge was the greatest predictor of Newborn Feeding Attitudes, being the physiological and emotional benefits of skin-to-skin contact for newborns and mothers and the indicators of effective suckling and milk transfer positive determinants of eating attitudes. Final-year students may have already had access to more training and experienced more clinical practices, which may have influenced the responses of our sample. Our results corroborate previous studies, which also found differences in the level of knowledge regarding newborn feeding among students in the health field, with those with more clinical experience scoring higher when compared to students in the early years of training [28,41,42].

Even more relevant were the significant results we obtained in the dimension of work practices that interfere with newborn feeding ability and, in this case, students in their first years of training (1st to 3rd years) scored higher. This dimension is directly related to practical experiences in the early stages of birth, which can negatively interfere with the newborn’s feeding skills. For example, in item 20 “Time required for skin-to-skin contact to breastfeed interferes with completion of required documentation” and item 18 “There is no time immediately after birth to allow uninterrupted skin-to-skin contact until the first breastfeed”. Furthermore, we found that work practices that interfere with newborn feeding ability were negatively correlated with and were a negative determinant of Overall Newborn Feeding Attitudes in our sample. Students at initial levels of training still did not have much contact with practice in specific areas, such as maternity and neonatal care, and this may have influenced the fact that the responses of participants at initial levels scored higher in this dimension [28,41,42]. Likewise, Vandewark [43] compared the level of knowledge of nursing students at junior and senior levels, and despite the fact that both groups were aware of the benefits and physiology of breastfeeding, knowledge about breastfeeding management was significantly higher in the group of more advanced students.

Thus, our results indicate that the level of knowledge directly influenced attitudes towards Newborn Feeding among our sample. These results have already been pointed out by other studies, in which having more knowledge about newborn nutrition was associated with having more positive attitudes towards it, which could help to promote the health of babies and better health and feeding practices among caregivers. On the other hand, less knowledge generates attitudes that can bring negative results, such as divergent and conflicting information between health professionals, which can confuse families and instill bad practices that can be harmful to the newborn [20,22,28,44,45].

In addition to these findings, it is necessary to analyze our results in a broader approach because, as far as we know, this study was the first to assess newborn feeding knowledge and attitudes among Portuguese medical students. Our global sample had a moderate score for all study variables, indicating average knowledge and attitudes about newborn feeding, as seen in other studies [19,46]. These results are important, as they address a gap in medical training in Portugal, and this could have potentially negative long-term implications as medical students, as future medical doctors, will often be at the forefront of nutrition care of newborns. In addition, regardless of the area of specialization, medical students and professionals are also frequently asked for health advice and consulting on best feeding practices [36,44,47,48,49].

Thus, the main contribution of this study is to corroborate the discussion about newborn feeding among medical students and to raise awareness regarding the urgent need for optimal newborn feeding practices to be taught in all stages of medical education from the early years of teaching [16,50,51]. In this sense, academic and health education institutions that promote a culture of learning and offer learning programs, either as part of the academic curriculum, through workshops, seminars, observation internships, or even as an online module, which is currently an important source of dissemination of knowledge [19,52,53,54,55,56], could be particularly effective in promoting a change in this scenario.

We suggest the creation of a learning/teaching culture that cultivates dynamic features to encourage younger students to understand the feeding processes of newborns and their consequences in a practical way. This is because practice, even in the classroom context, can be effective when addressing potential real cases. Thus, experienced techniques that encourage students to apply theoretical knowledge, such as role-plays and storytelling, can be useful strategies for consolidating learning. Moreover, encouraging students to actively participate in classroom discussions and making sure they are active listeners and contribute their own ideas through formal discussion can be beneficial for them to understand complex topics more easily. This would help prepare future health professionals to guide families about the benefits and risks of different types of nutrition, as well as provide the necessary skills to recognize and treat complications related to this process [57,58].

In addition, we point to the need for these learnings to address taboos related to newborn feeding, such as the different contexts and settings in which families are involved. As seen before, there are different factors that can influence the newborn’s feeding practice, such as socioeconomic level, family size, work, and belief systems, among others [8,9,10,59,60]. The discussion about newborn feeding for medical students is even more relevant when dealing with family situations that are not commonly presented, such as the challenges of single-father families created by surrogacy [61], women with HIV [62,63], diabetes, or in the case of mothers who are unwilling or unable to breastfeed their babies [59,64]. These families may need different approaches and different recommendations from the medical team.

Hence, in addition to knowing adequate practices, it is necessary for future health professionals to be able to act effectively with the family idiosyncrasies presented to them and to be able to make appropriate recommendations for each case [65]. For this, it is important that educators are also prepared and know the basic guidelines on feeding babies, and that they can pass them on to future professionals. Again, we suggest that this may be based on the need for constant updating of knowledge on the part of professors who address these topics and on reviewing their previous experiences. Moreover, learning from other countries and authors may be useful, so they can be familiar with the different social, cultural, and local realities, as well as with the potential problems that people can face during the practice of feeding a newborn.

However, despite these important contributions, it is important to note certain limitations of the study. The first one is related to the characteristics of the sample. The sample is small when compared to the number of medical students enrolled in Portugal on the date of the study (N = 12,575). In addition, about 85% of the sample came from 03 of the 08 Portuguese universities that offer medical training programs [66], raising some questions regarding the sample’s representativeness in comparison with the national results. Furthermore, the cross-sectional nature of the study also posed a limitation, as the knowledge is a variable constructed and altered over time [42]. Another limitation is due to the fact that knowledge and attitudes about newborn feeding are composed of different variables that may not have been covered by the measures that were used. Finally, given that the questionnaire was both made available online and self-administered, these study characteristics raise questions regarding the possible influence of selection bias.

In order to address these limitations, we suggest that future studies use larger and more representative samples to provide more generalizable and accurate estimates of the data. Moreover, future longitudinal and face-to-face studies could also improve the understanding regarding the process of newborn feeding knowledge and attitudes in medical students over years of study. Health professionals’ own beliefs and experiences regarding breastfeeding and newborn feeding can influence their practice of working with families [16,17,18,19]. In this sense, we recommend the incorporation of additional measures in future studies, such as the evaluation of personal belief systems and personality style, student interest in the area, experiences with newborns and the initial feeding process. The inclusion of these and other variables could assist in verifying additional predictive and preventive factors that may influence the attitudes of future doctors. In addition, further research should be conducted among other health students, such as nursing and midwifery, in order to understand these results in the different categories of health education in the country. A database composed and updated on the learning and potential difficulties of medical students in relation to the feeding of newborns is also needed so that the necessary changes can be proposed and implemented.

Finally, we reaffirm the need for further studies on this topic in Portugal, which is still scarce. Although our results indicate that there are differences in students’ knowledge depending on the year of training, future studies should seek to understand the relationship, the deep meaning, and implications of this difference in order to propose more effective actions to improve teaching at different levels of training.

## Figures and Tables

**Table 1:** Overall results for all variables under study.

	M	SD
Physiological and emotional benefits of skin-to-skin contact for newborns and mothers	3.76	0.50
Indicators of effective suckling and milk transfer	3.51	0.63
Work practices that interfere with newborn feeding ability	3.03	0.37
Overall Newborn Feeding Knowledge	3.39	0.36
Overall Newborn Feeding Attitudes	3.81	0.41

Note: M—mean; SD—standard deviation.

**Table 2:** Results for all variables by gender.

		M	SD	t(df)	*p*
Physiological and emotional benefits of skin-to-skin contact for newborns and mothers	Women	3.79	0.49	3.337(647)	0.001 *
	Men	3.63	0.52		
Indicators of effective suckling and milk transfer	Women	3.52	0.62	1.318(647)	0.188
	Men	3.45	0.65		
Work practices that interfere with newborn feeding ability	Women	3.03	0.37	−1.137(647)	0.256
	Men	3.07	0.37		
Overall Newborn Feeding Knowledge	Women	3.40	0.35	1.587(647)	0.113
	Men	3.35	0.39		
Overall Newborn Feeding Attitudes	Women	3.81	0.41	0.162(647)	0.871
	Men	3.80	0.42		

Note: M—mean; SD—standard deviation; t(df)—t student (degrees of freedom); *p*—probability value. * < 0.05.

**Table 3:** Results for all variables by year of training.

		M	SD	F	*p*
Physiological and emotional benefits of skin-to-skin contact for newborns and mothers	1st	3.71	0.53	1.130	0.343
	2nd	3.71	0.51		
	3rd	3.70	0.48		
	4th	3.78	0.49		
	5th	3.82	0.51		
	6th	3.80	0.47		
Indicators of effective suckling and milk transfer	1st	3.41	0.65	2.614	0.024 *
	2nd	3.40	0.69		
	3rd	3.52	0.63		
	4th	3.49	0.57		
	5th	3.64	0.62		
	6th	3.58	0.58		
Work practices that interfere with newborn feeding ability	1st	3.15	0.36		
	2nd	3.07	0.42	3.637	0.003 *
	3rd	3.02	0.37		
	4th	2.99	0.29		
	5th	2.99	0.34		
	6th	2.98	0.39		
Overall Newborn Feeding Knowledge	1st	3.40	0.37	0.416	0.838
	2nd	3.36	0.42		
	3rd	3.37	0.33		
	4th	3.37	0.31		
	5th	3.42	0.33		
	6th	3.40	0.35		
Overall Newborn Feeding Attitudes	1st	3.59	0.41	16.549	0.000 **
	2nd	3.70	0.38		
	3rd	3.82	0.44		
	4th	3.79	0.37		
	5th	3.96	0.39		
	6th	3.98	0.34		

Note: M—mean; SD—standard deviation; F—ANOVA; *p*—probability value. * < 0.05; ** < 0.001.

**Table 4:** Correlation Matrix.

	1	2	3	4	5	6	7
1—Age	1						
2—Year of training	0.475 **	1					
3—Physiological and emotional benefits of skin-to-skin contact for newborns and mothers	0.165 **	0.084 *	1				
4—Indicators of effective suckling and milk transfer	0.152 **	0.124 **	0.592 **	1			
5—Work practices that interfere with newborn feeding ability	−0.188 **	−0.151 **	0.197 **	0.274 **	1		
6—Overall Newborn Feeding Knowledge	0.057	0.024	0.800 **	0.815 **	0.649 **	1	
7—Overall Newborn Feeding Attitudes	0.235 **	0.327 **	0.303 **	0.302 **	−0.140 **	0.204 **	1

Note: * < 0.05; ** < 0.001.

**Table 5:** Hierarchical linear regression analysis predicting Overall Newborn Feeding Attitudes.

	Model I			Model II		
	B	SE B	β	B	SE B	β
Age	0.010	0.004	0.102 *	0.002	0.004	0.017
Gender	−0.031	0.037	−0.031	0.019	0.035	0.019
Year of training	0.066	0.010	0.281 **	0.057	0.009	0.246 **
Physiological and emotional benefits of skin-to-skin contact for newborns and mothers				0.164	0.036	0.199 **
Indicators of effective suckling and milk transfer				0.134	0.029	0.205 **
Work practices that interfere with newborn feeding ability				−0.218	0.042	−0.196 **
R^2^	0.116			0.234		
F	28.307 **			32.692 **		

Note: B—unstandardized beta; SE B—standard error for unstandardized beta; β—*t* test statistic; R^2^—Coefficient of Determination; F—value obtained from a regression analysis to compare means of two populations. * < 0.05; ** < 0.001.

## Data Availability

The data presented in this study are available upon request.
